# Type I interferon pathway activation in connective tissue disease associated interstitial lung disease

**DOI:** 10.3389/fimmu.2026.1845177

**Published:** 2026-06-11

**Authors:** Tobias M. Defesche, Thomas Koudstaal, Marjan A. Versnel, Odilia B.J. Corneth, Zana Brkic

**Affiliations:** 1 Department of Immunology, Erasmus Medical Centre, Rotterdam, Netherlands; 2 Department of Internal Medicine, Division of Clinical Immunology and Allergology, Erasmus Medical Centre, Rotterdam, Netherlands; 3 Department of Pulmonary Medicine, Erasmus Medical Centre, Rotterdam, Netherlands

**Keywords:** autoimmunity, connective tissue disease (CTD), fibrosis, interstitial lung disease (ILD), type I interferon (IFN-I)

## Abstract

Interstitial lung disease (ILD) underlies morbidity and mortality in connective tissue diseases (CTDs) such as systemic sclerosis. ILD encompasses a range of inflammatory and fibrotic pneumopathies, some of which manifest progressive characteristics. Up to 40% of ILDs are progressive, marked by radiologic progression, lung function decline, and increased symptoms. Immunosuppression is standard therapy for ILD, whereas antifibrotics like nintedanib are used in progressive fibrotic cases. Many patients deteriorate despite treatment, underscoring the need for biomarkers and targeted interventions. Type I interferon (IFN-I) signaling is implicated in CTD-ILD: interferon stimulated gene expression correlates with accelerated pulmonary decline and sustained IFN-I can precipitate ILD. In addition, IFN-I blocking agents like anifrolumab are under clinical investigation for CTD-ILD. This review examines IFN-I’s role and therapeutic targeting in ILD.

## Highlights

- IFN-I activation is implicated in CTD-ILD development.
- In CTD-ILD, FVC decline parallels increased IFN-I signaling, suggesting a role in patient care.
- Early IFN-I modulating therapy studies in CTD-ILD show promising results.

## Introduction

### Clinical ILD characteristics

Interstitial lung disease (ILD) comprises a heterogeneous group of disorders characterized by inflammation and/or fibrosis of the pulmonary interstitium, leading to reduced compliance and impaired gas exchange (1, 2). Connective tissue disease (CTD), most notably systemic sclerosis (SSc), rheumatoid arthritis (RA) and idiopathic inflammatory myopathies (IIMs), account for 25–30% of all ILD and up to 40% of progressive pulmonary fibrosis (PPF) cases (3). Within these CTDs, ILD is identified in 10–35% of RA patients (4, 5), is present at baseline in 34–53% of SSc patients (6), and affects roughly 41% of IIM patients (7). Additionally, ILD is also prevalent in roughly 25% of patients suffering from Sjögren’s Disease (SjD) (8).

Disease course varies highly, ranging from indolent to rapidly progressive even within the same CTD (9). Serial pulmonary function testing defines progression of ILD in CTD as ≥ 10% relative decline in forced vital capacity (FVC), 5-10% reduction in FVC combined with ≥ 15% decline in diffusing capacity for carbon monoxide (DLCO) or as radiological progression (10, 11).

**Figure 1:**
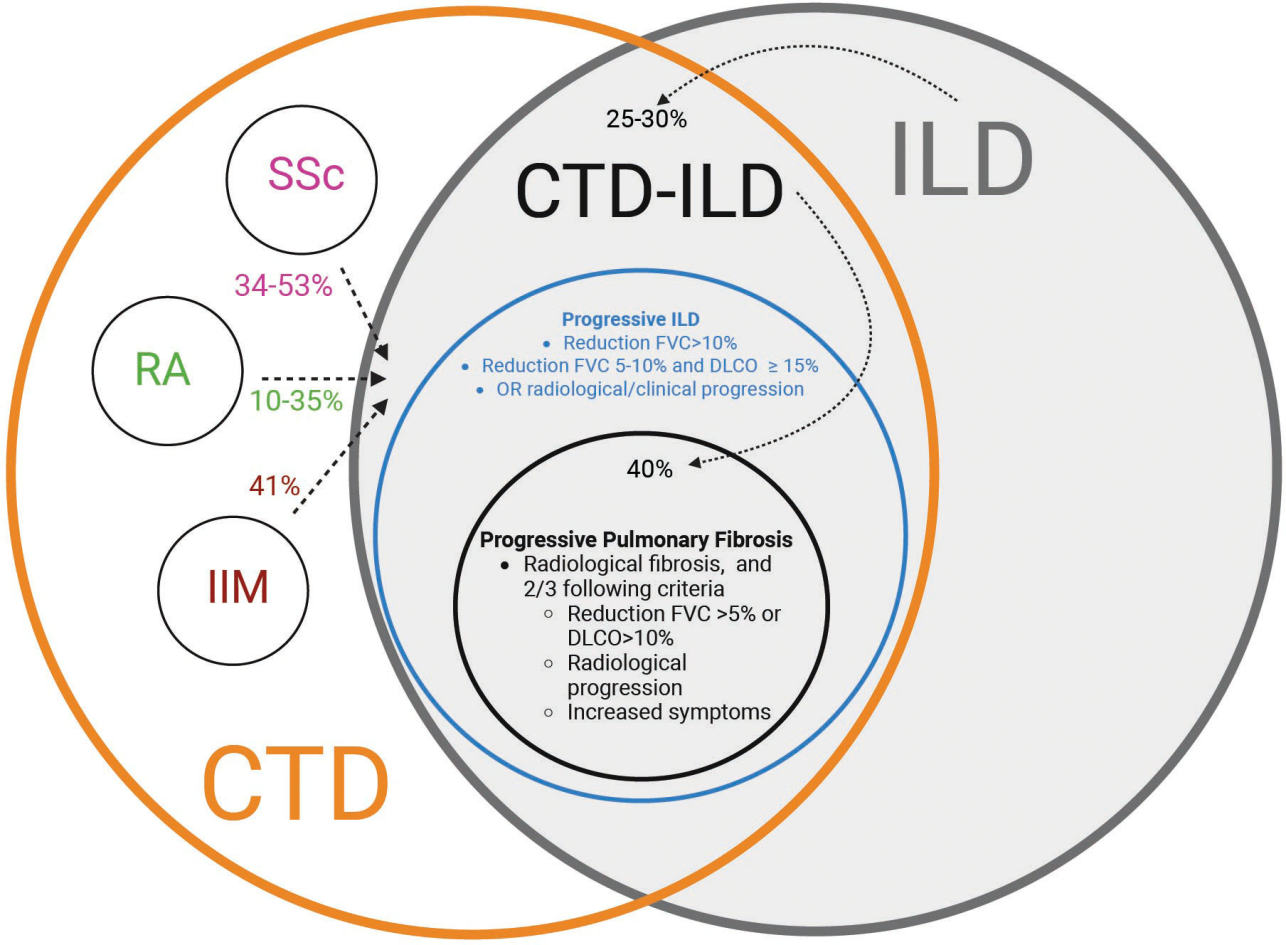
Visual overview of CTD-ILD subgroups within interstitial lung disease (ILD). A connective tissue disease (CTD) is identified as the underlying cause in approximately 25-30% of ILD cases. Among patients with CTD-ILD, an estimated 40% progress to a progressive pulmonary fibrosis (PPF) phenotype. Created in BioRender. Dik, W. (2026) https://BioRender.com/hq8l2ij.

In CTD-ILD, persistent alveolar inflammation triggers aberrant tissue repair, leading to interstitial scarring and development of pulmonary fibrosis. In a subset of patients, this fibrotic process acquires a self-sustaining progressive trajectory, culminating in PPF. PPF is diagnosed when pulmonary function test decline is accompanied by radiological increased fibrosis or increasing respiratory symptoms (12). Up to 40% of patients with CTD-ILD develop PPF, resulting in poor survival rates approaching those of idiopathic pulmonary fibrosis (IPF) (13). Rates of fibrosis and PPF are highly heterogeneous between different CTD-ILDs (8, 14–20). Diagnostic subgroups and their risk of progression are summarized in **Figure 1** and **Table 1**.

**Table 1:** Prevalence of ILD, fibrotic ILD and PPF in various CTD patient subgroups.

Underlying CTD	ILD prevalence (% of CTD)	Fibrosis prevalence on HRTC	PPF prevalence (% of ILD)	References
SSc	34-53%	Up to 80%	25-45%	Roeser et al. Respir Res. 2025 PMID 41188867 (15)
RA	10-35%	45-50%	30-50%	Mena-Vázquez et al. Diagnostics. 2021 PMID 34679492, Matteson et al. ACR Open Rheumatol. 2024 PMID 39243209, Sebastiani et al. J Clin Med. 2023, PMID 38002655 (16–18)
IIM	41%	Reports on fibrosis are lacking.*	18%	Zanatta et al. RMD Open 2023 PMID 37541742 (19)
SjD	23,7%	53-66%	36%	Manfredi et al. Semin Arthritis Rheum 2025 PMID 41442876, Manfredi et al. J Pers Med 2024 PMID 39063962 (20)

*In one cohort, usual interstitial pneumonia (UIP, by definition fibrotic) was prevalent in 28% of patients. Possibly total prevalence is higher as fibrosis is not exclusive to UIP.

CTD-ILDs are treated depending on their underlying CTD as prescribed by current EULAR guidelines, reflecting their differences in pathobiology (21–23). Across CTDs, antifibrotic therapy is reserved for patients who demonstrate progressive fibrosis despite adequate immunomodulation. This treatment is consistent with the 2022 ATS/ERS guideline for PPF (12). Despite increasingly disease-specific strategies, many patients continue to progress, underscoring the need for mechanism based biomarkers and targeted treatments (9).

## IFN-I biology in ILD

Type I interferons (IFN-I), mainly IFN-α and IFN-β, are cytokines produced by various cells, primarily plasmacytoid dendritic cells (pDCs), in response to viral infection. Viral nucleic acids like double-stranded RNA or CpG-rich DNA are detected by pDCs through pattern recognition receptors, including Toll-like receptor (TLR) 7, retinoic acid-inducible gene-I-like (RIG-I) receptors, and cyclic GMP-AMP synthase (cGAS). Detection triggers intracellular signaling cascades that activate transcription factors like interferon regulatory factor (IRF) 3 and IRF7, which drive transcription and secretion of IFN-I (24). Once secreted, IFN-I binds the type I interferon receptor (IFNAR), composed of IFNAR1 and IFNAR2 subunits. Virtually all nucleated cells express IFNAR and are thus IFN-I-responsive. Binding of IFN-I to IFNAR activates the JAK-STAT signaling pathway, leading to expression of hundreds of interferon-stimulated genes (ISGs) that limit viral replication by degrading viral RNA, inhibiting translation, and promoting apoptosis of infected cells (25).

Beyond antiviral roles, IFN-I has broad immunomodulatory functions. It enhances antigen presentation by upregulating MHC-I on most antigen presenting cells and MHC-II and costimulatory molecules on dendritic cells (DCs), improving their T-cell activating capacities. IFN-I directly supports CD8+ T-cell responses by promoting their expansion, survival, and cytotoxic activity, including increased production of perforin and granzyme B (26). In CD4+ T-cells, IFN-I promotes differentiation into Th1 cells by enhancing responsiveness to IL-12 and increasing expression of the transcription factor T-bet. IFN-I influences B-cells by promoting their activation, antibody production, and class-switch recombination, particularly toward IgG isotypes (26).

IFN-I has been implicated in CTD-ILD. Exogenous IFN-α therapy or monogenic interferonopathies, in which IFN-I signaling is overactive (STING-associated vasculopathy with onset in infancy, SAVI) can lead to ILD (27–30). Furthermore, in SSc, the degree of IFN-I pathway activation correlates with disease severity (31–33). ILD disproportionately affects severe CTD (3), further implicating IFN-I in ILD pathophysiology. Additionally, IFN-I activation correlates with accelerated FVC decline in CTD-ILD (32–34). Taken together, these observations indicate a role for IFN-I in CTD-ILD and point to IFNAR blockade as a therapeutic strategy for CTD-ILD. Anifrolumab, an anti-IFNAR monoclonal antibody, FDA approved for systemic lupus erythematosus (SLE), is in phase 3 evaluation for SSc-ILD (DAISY trial, NCT05925803 I), with Revised-CRISS-25 score (which encompasses FVC-% predicted) as primary endpoint (35). Current reports on the effects of anifrolumab on ILD in SLE are lacking.

In this review we will summarize and discuss the clinical and genetic evidence that excessive IFN-I can trigger or accelerate ILD, explore how sustained IFN-I signaling remodels endothelium, epithelium, macrophages and fibroblasts, and evaluate interferon-targeted treatments.

The literature search was conducted in Medline, Embase, Web of Science Core Collection, and the Cochrane Central Register of Controlled Trials using comprehensive interferon-related terms including type I interferons (‘IFN-I’, ‘IFNα’, ‘IFNβ’), related therapies (‘anifrolumab’, ‘JAK inhibition’, ‘peg-IFN’) combined with ILD terms and synonyms (‘interstitial lung disease’, ‘interstitial pneumonitis’, ‘interstitial pulmonary disorder’). Google Scholar was additionally searched (top 100 ranked records) using IFN-I and ILD term combinations. The search was limited to articles written in English and excluded conference abstracts. Additional relevant publications identified through reference lists of included studies were also considered for inclusion. Articles were subject to three rounds of selection based on relevance (title, abstract, full text).

## IFN-I driven ILD

Evidence from case reports and genetic studies demonstrates that IFN-I, administered therapeutically or constitutively expressed due to genetic mutations, contributes to ILD pathogenesis. This connection is supported by associations between *IRF* polymorphisms and ILD, highlighting the importance of the IFN-I in ILD.

### Exogeneous administration of IFN-I

Several case reports describe ILD following therapeutic IFN-α, usually peg-IFN-α2b used for chronic hepatitis B or oncologic indications. Symptoms develop within 2–24 weeks and include dry cough, dyspnea, and reduced exercise tolerance. High-resolution CT (HRCT) often shows ground-glass opacities, reticular patterns and pulmonary infiltrates. In cases where bronchoalveolar lavage is performed, a lymphocytic predominance is demonstrated (27, 28). Discontinuation of IFN-I treatment, sometimes supported by corticosteroids, usually leads to improvement although residual radiologic abnormalities may persist.

### Endogenous overproduction of IFN-I

**Figure 2:**
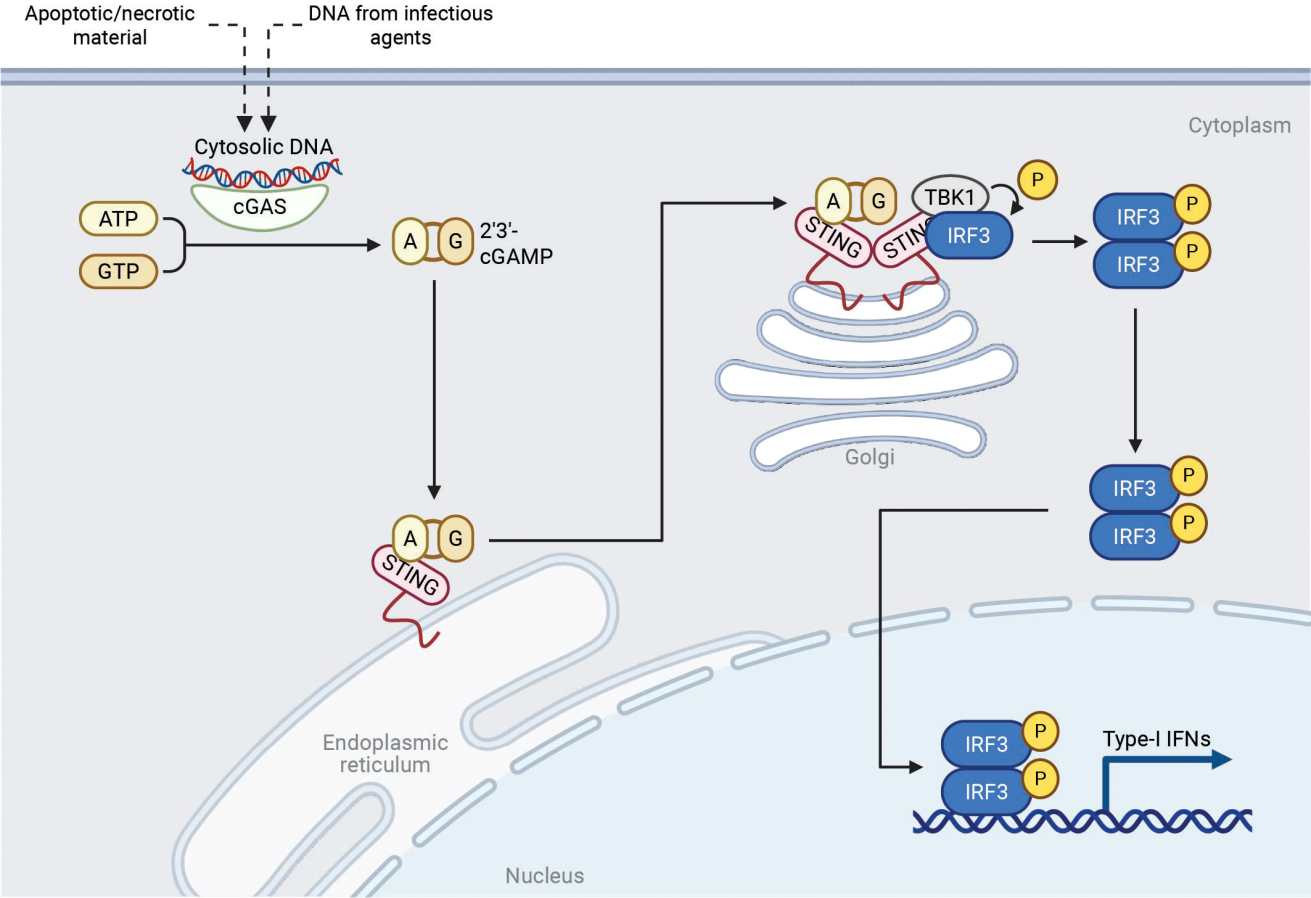
Schematic overview of cGAS-STING signaling cascade triggered by cytosolic DNA, eventually resulting in production of IFN-I. Created in BioRender. Defesche, T. (2026) https://BioRender.com/r8gw001.

In healthy individuals, IFN-I production occurs when cGAS is triggered by cytosolic DNA to produce cyclic dinucleotides, which activate stimulator of interferon genes (STING) to translocate to the Golgi. This induces downstream signaling through TANK-binding kinase 1 (TBK1) and IRF3 mediated IFN-I expression (**Figure 2**). Monogenic interferonopathies illustrate the pathogenic potential of constitutive IFN-I activation. In SAVI, a gain of function mutation in *TMEM173* (encoding STING protein) drives ligand-independent activation of the cGAS–STING–TBK1–IRF3 axis, resulting in persistent IFN-I transcription (29, 30). Patients develop cutaneous vasculopathy, systemic inflammation, and almost universally ILD. This may be due to the fact that STING is physiologically expressed in type 2 pneumocytes, bronchial epithelium and alveolar macrophages. Expression of the mutated STING protein in these cells increases IFN-I expression, making it the likely culprit for ILD development (29). Accordingly, targeted therapies with Janus kinase inhibitors (JAKis) or anifrolumab have shown improvement of ILD symptoms along with decreased ISG expression (36). Of note, JAKis commonly used in these diseases (tofacitinib, baricitinib) do not only affect IFN-I signaling, but also affect downstream signaling of other cytokines such as IL-6 (37).

A different source of endogenously produced IFN-I are viral infections. Currently, there is no evidence linking viral infections to the onset of ILD in CTD. However, in existing CTD-ILD, concomitant viral infection can significantly worsen disease (38). Although the study does not directly measure IFN-I pathway activation, this could indicate that virally induced IFN-I is a pathogenic factor in CTD-ILD.

### Genetic susceptibility in CTD-ILD

IRF polymorphisms, particularly of *IRF5*, are associated with ILD in SSc (39–41). Upon phosphorylation by, for example, TBK1, IRFs change shape and translocate to the nucleus and promote transcription of IFN-I (42). Although it is unclear whether these polymorphisms increase or decrease the amount of IFN-I, the findings do indicate involvement of IFN-I signaling in SSc-ILD. Interestingly, *IRF5* also regulates IL-6 expression through binding of the IL-6 promotor. Activating polymorphisms in *IRF5* could concomitantly upregulate IFN-I and IL-6 in patients. In SSc-ILD patients, upregulation of IL-6, a critical cytokine in inflammation and fibrosis, is present and inversely correlates with DLCO (43).

## IFN-I effects on key cellular players in CTD-ILD

ILD development in CTD originates from injury to the pulmonary vascular endothelium or the alveolar epithelium (44–47). Endothelial damage arises from CTD-associated vasculopathy, driven by autoimmune-mediated destruction of vascular endothelium, whereas epithelial injury is triggered by infections, autoimmune attack, oxidative stress secondary to vasculopathy, or mechanical insults such as micro-aspiration of gastric contents (44).

These initial injuries activate tissue-resident innate immune cells, including pDCs and monocytes, which respond by releasing large amounts of IFN-I, facilitating recruitment of innate cells like neutrophils (46). Activation of this innate immune network amplifies inflammation, promoting recruitment of additional innate and adaptive immune cells. This cascade sustains cytokine release and autoantibody production, resulting in further epithelial and endothelial damage and establishing a cycle of persistent inflammation (46).

Chronic inflammation drives fibroblast proliferation, migration, and differentiation into myofibroblasts, which secrete extracellular matrix (ECM) components (e.g. collagen), ultimately leading to progressive fibrosis (46).

**Figure 3:**
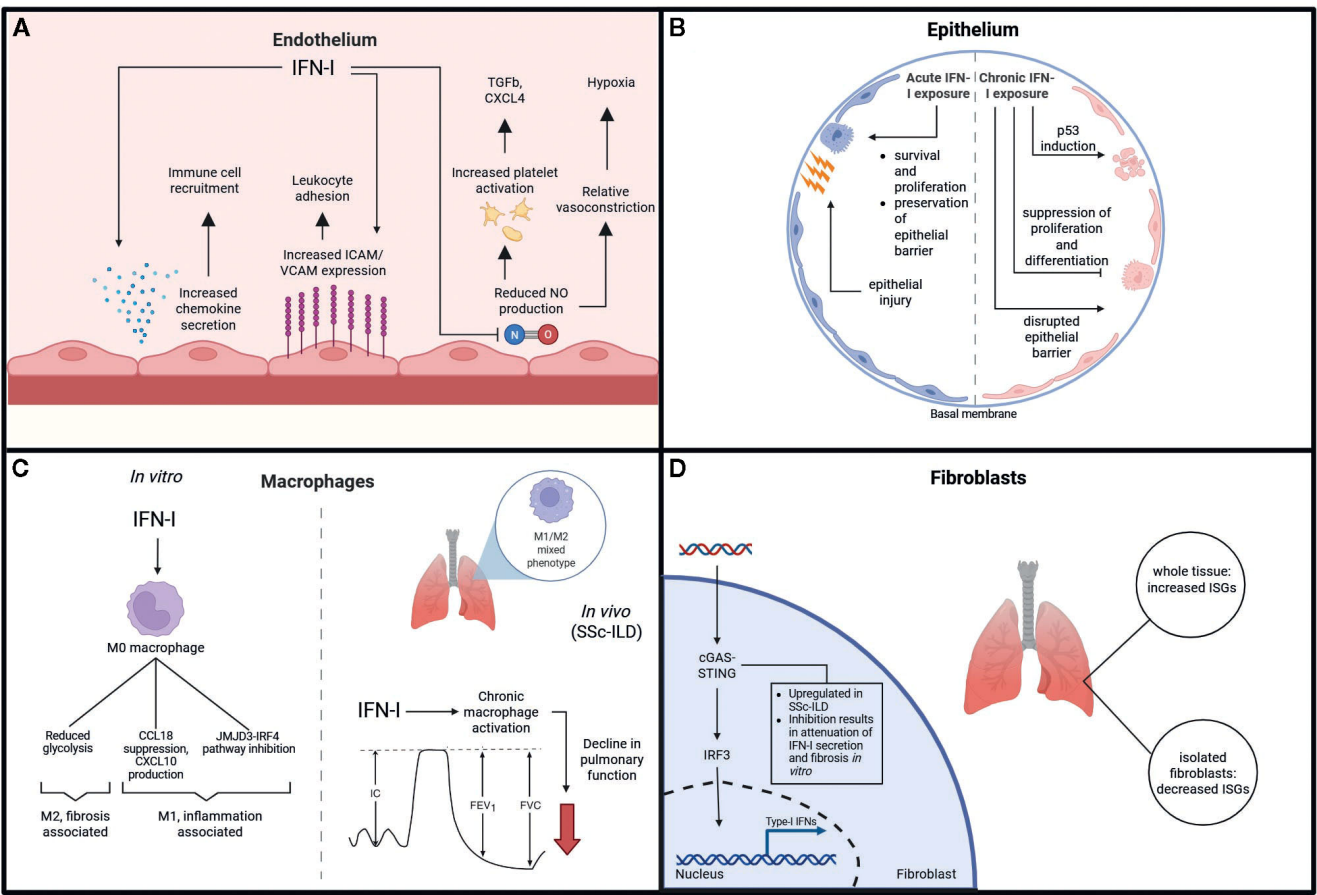
Overview of the effects of IFN-I on various cell types. **(A)** Endothelium: IFN-I induces immune cell recruitment through chemokine secretion. Also, IFN-I induces expression of adhesion molecules, facilitating adhesion and diapedesis. IFN-I inhibits NO production resulting in vasoconstriction and subsequent hypoxia, and platelet activation and subsequent release of TGFb and CXCL4. **(B)** Epithelium: acute IFN-I exposure promotes survival and proliferation, restoring the epithelial barrier, chronic IFN-I exposure promotes apoptosis and inhibits survival and proliferation, disrupting the epithelial barrier. **(C)** Macrophages: *in vitro*, macrophages acquire both M1 and M2 characteristics upon IFN-I stimulation. *In vivo*: a mixed M1/M2 phenotype is found in SSc-ILD patients. In SSc-ILD patients, IFN-I signaling correlates with macrophage activation and pulmonary functions decline. **(D)** Fibroblasts: cGAS-STING is upregulated is SSc-ILD pulmonary fibroblasts, inhibition attenuates IFN-I secretion and fibrosis *in vitro*. Expression of IFN-I stimulated genes is increased pulmonary tissue as a whole but decreased in fibroblasts, in SSc-ILD. Created in BioRender. Dik, W. (2026) https://BioRender.com/thbs8ky.

IFN-I influences multiple steps in this cascade, acting on endothelial cells, epithelial cells, and innate and adaptive immune populations to promote inflammation and tissue remodeling. In the following sections, we discuss the effects of IFN-I on cell types involved in CTD-ILD and how these interactions contribute to disease progression. A summary of mechanisms is shown in **Figure 3**.

### Endothelial cells

Endothelial homeostasis is crucial for a healthy interstitium, but is often impaired in CTD-ILD. Several lines of evidence support a role for IFN-I in lung endothelial disruption. Single-cell RNA-seq of microvascular endothelial cells (MVECs) isolated from explanted SSc-ILD lungs revealed upregulation of ISGs and other antiviral transcripts compared with healthy MVECs (48). Furthermore, in SAVI, patients not only develop ILD but also present with pronounced cutaneous and systemic vasculopathy (29, 36). These vascular symptoms likely follow from IFN-I signaling. This is indicated by the fact that conditioned media, likely containing large amounts of IFN-I, from leucocytes with an activating STING mutation (as seen in SAVI) was directly toxic to endothelial cells (49).

Circulating biomarkers reinforce this link. In CTD-ILD cohorts, serum Galectin-9, a protein strongly but not exclusively IFN-I induced, correlates with endothelin-1 (ET-1), a marker of endothelial damage (50). In SLE, markers of endothelial dysfunction like the presence of soluble VCAM-1 and endothelial-derived microparticles correlate with IFN-I signature scores (a composite score of ISG expression) (51).

Mechanistic data show that IFN-I can drive endothelial pathology. In pulmonary endothelial cells, IFN-I induces CX3CL1 and CCL5, contributing to leukocyte recruitment and vascular inflammation (52). Moreover, IFN-I upregulates expression of adhesion molecules ICAM-1 and VCAM-1 to enhance trans-endothelial migration of leucocytes towards underlying tissues where they contribute to inflammation (53, 54).

In the endothelium, nitric oxide (NO) is produced by endothelial nitric oxide synthetase (eNOS). NO promotes vasodilation and angiogenic repair, and inhibits platelet adhesion, activation and release of profibrotic mediators such as TGF-β and CXCL4. Interestingly, CXCL4 levels are elevated in SSc-ILD and fall with immunosuppression (55). Immune complexes (ICs) containing CXCL4 and nucleic acids stimulate IFN-I production by pDCs (56, 57). IFN-I suppresses eNOS transcription *in vitro*: exposure of human umbilical vein endothelial cells (HUVECs) to IFN-I lowers NO production. Subsequently, this leads to relative vasoconstriction, impaired angiogenic repair and increased platelet adhesion and activation (58, 59).

In summary, endothelial damage initiates IFN-I production, while IFN-I in turn exacerbates endothelial dysfunction, setting up a self-perpetuating cycle of vascular injury.

### Epithelial cells

In CTD-ILD, autoimmune and environmental triggers cause immune-mediated injury to the alveolar epithelium, leading to epithelial dysfunction, senescence, and impaired repair. In some patients this defective epithelial regeneration then drives persistent activation of fibroblasts and myofibroblasts, excessive ECM deposition and ultimately chronic interstitial fibrosis (60). Functioning of the alveolar epithelium is influenced by IFN-I signaling. In murine models of acute sterile injury (bleomycin) or Pseudomonas pneumonia, transient IFN-I signaling limits neutrophil-driven damage and tightens epithelial junctions, thereby reducing collagen deposition and post-inflammatory leakiness (61). Similar barrier protective effects are found in Streptococcus pneumoniae infection, where exogenous IFN-β prevented bacterial transmigration across the lung by upregulating tight junction proteins (62).

However, prolonged IFN-I signaling disrupts epithelial repair by inducing p53, suppressing proliferation, and impairing differentiation, compromising regeneration of the epithelial barrier and increasing risk of secondary infections and fibrosis (63).

These disruptive effects of IFN-I on epithelial proliferation is reinforced by the finding that knocking out *IFNAR1, IFNAR2, JAK1* or *TYK2* in SARS-CoV-2 infected epithelial cells results in increased proliferation. Additionally, prolonged exposure to exogeneous IFN-I, without the presence of viral infection, reduces proliferation in iPSC-derived type 2 alveolar epithelial cells (64). Reports on the effects of IFN-I on type 1 alveolar cells in the context of CTD-ILD are lacking.

Together, these findings indicate an effect of IFN-I on pulmonary epithelium that depends on the duration of exposure.

### Macrophages

Macrophages, particularly alveolar and interstitial subsets, are drivers of inflammation and fibrosis. Alveolar macrophages reside in the pulmonary alveoli and are primarily tissue-resident cells derived from fetal monocytes (65). Pulmonary interstitial macrophages form a more heterogeneous population that includes tissue-resident and monocyte-derived cells (66).

In CTD-ILD pulmonary tissue, macrophages display complex activation states that display characteristics of type 1 (pro-inflammatory) and type 2 (tissue repair, resolution) phenotypes. These macrophages co-express inflammatory markers like MHC-II and tissue-repair markers such as CD163, and show upregulation of genes involved in cytokine signaling, antigen presentation, and matrix remodeling. This suggests that macrophages are transcriptionally primed for rapid, context-dependent responses (67). Additionally, in SSc-ILD, a phenotypical shift from a type 1 phenotype towards a type 2 phenotype has been implicated in fibrotic progression of pulmonary disease (68).

IFN-Is modulate the macrophage phenotype. *In vitro*, several effects of IFN-I on macrophage polarization are described. Two studies found glycolysis, which is associated with M1-like proinflammatory phenotypes, to be decreased in IFN-I stimulated macrophages (69, 70). Another study described the inhibitory effect of IFN-I on the JMJD3-IRF4 pathway, a downstream effector of IL-4 and highly associated with M2 polarization (71). Inhibition of the M2 phenotype, which is associated with resolution of inflammation, contributes to chronic inflammation. Contrary to these findings, a recent study found IFN-I stimulation to expand efferocytotic capacities in macrophages, which is classically associated with the M2 phenotype (72).

Perhaps this indicates a role for IFN-I beyond classical M1/M2 thinking in CTD-ILD, as is in line with recent studies indicating more mixed phenotypes *in vivo* in IPF (73–75).

In CTD-ILD, lung involvement is often thought to begin as an inflammatory interstitial process, and in a subset of patients this persistent immune-mediated injury transitions over time into a predominantly fibrotic phenotype (76). IFN-I drives chronic immune activation and subsequent fibrosis through chemokine production via macrophage and DC pathways, as demonstrated in a murine model of pulmonary fibrosis (77). These findings are consistent with prior work by Christmann et al., who showed that SSc-ILD pulmonary tissue exhibits co-activation of IFN-I-regulated genes and macrophage activation pathways correlating with lung function decline and radiographic severity (78). However, in a co-culture of alveolar epithelial cells and macrophages IFN-I induces production of CXCL10 and suppresses production of fibrotic mediators such as CCL18 (79). CCL18 is particularly relevant in CTD-ILD as elevated levels in serum and bronchoalveolar lavage fluid are associated with fibrotic progression and poor prognosis (80, 81).

While IFN-I may limit fibrosis under certain conditions, together, these studies underscore the context-specific and potentially targetable role of IFN-I driven macrophage programming in CTD-ILD.

### Fibroblasts

Differentiation of fibroblasts into myofibroblasts is crucial in development of ILD and pulmonary fibrosis. Pulmonary myofibroblasts contribute to disease through excessive ECM production and secretion of inflammatory mediators (82). Classically, this differentiation is triggered by TGF-β, which is released during tissue injury by platelets, macrophages, and epithelial cells (83). In CTDs, chronic immune activation results in persistent tissue damage and continuous TGF-β signaling, leading to myofibroblast accumulation and fibrosis (84).

There seems to be cross-talk between IFN-I and TGF-β signaling. Treatment with IFN-I in a murine bleomycin model of pulmonary fibrosis lowers protein levels of TGF-β and subsequent pulmonary fibrosis (85).

Recent studies suggest that pathways such as cGAS-STING also contribute to fibroblast activation in SSc-ILD. *In vitro*, dual stimulation of fibroblasts with TGF-β and DNA fragments induces a myofibroblast phenotype resembling those found in SSc-ILD, characterized by increased alpha smooth muscle actin (α-SMA) and IFN-I secretion. Additionally, fibroblasts derived from SSc-ILD lungs exhibit upregulated cGAS expression at both mRNA and protein levels, and inhibition of the cGAS-STING pathway attenuates their dual pro-fibrotic and pro-inflammatory phenotype (86).

However, the role of IFN-I signaling appears to be complex. While lung tissue in SSc-ILD shows elevated IFN signatures, lung fibroblasts isolated from these patients show reduced expression of ISGs (86). This suggests that fibroblasts show increased IFN-I production, while being less responsive to IFN-I themselves, possibly due to negative feedback loops resulting in receptor downregulation. It might also indicate that IFN-I promotes fibrosis indirectly via immune cell activation while exerting negative feedback on fibroblasts themselves.

## IFN-I in specific ILD subtypes

CTD-ILD most commonly arises in the context of DM and SSc but also in diseases such as SjD and SLE. In RA, ILD is also prevalent and a leading cause of mortality. Although literature is limited for RA-ILD (as is true for SjD-ILD) (**Table 1**), a role for IFN-I is suggested as CXCL10, an IFN-I induced chemokine, is present at higher levels in RA-ILD patients compared to non-ILD RA patients (87, 88). Predicting ILD development in these diseases remains challenging. However, the presence of specific autoantibodies (anti-MDA5 in DM, anti-Scl70 in SSc) is a strong risk factor. When these autoantibodies are present in ICs and taken up by pDCs, they potently induce IFN-I production *in vitro* (89, 90). Though CTD-ILD is very heterogeneous, general recommendations can be found in the Points for clinical practice (Box 1).

### Box 1 – Points for clinical practice

- IFN-I pathway activation is a key feature in SSc- and anti-MDA5+DM-ILD. Elevated IFN-I signatures correlate with increased disease activity and faster FVC decline. Recognizing IFN-I involvement can help guide disease severity assessment and risk stratification in SSc and anti-MDA5+DM, and likely other CTD-ILDs.
- Measuring ISG as a surrogate marker for IFN-I expression in blood or lung tissue emerges as a promising biomarker approach for disease activity and therapeutic response prediction. Such precision tools are becoming increasingly relevant for tailoring immunomodulatory treatment strategies in CTD-ILD patients.
- Treatment decisions increasingly incorporate targeting IFN-I signaling pathways. JAKis show therapeutic promise in controlling IFN-I driven inflammation in patients with aggressive or refractory CTD-ILD. Conventional immunosuppressive agents remain fundamental but may be augmented or personalized based on IFN-I pathway activity.

### IFN-I in anti-MDA5 dermatomyositis–associated ILD

In anti-MDA5 positive DM-ILD (MDA5+ DM-ILD), pathogenesis is thought to begin with ICs composed of anti-MDA5 antibodies and self-RNA. These complexes are internalized by pDCs via Fcγ receptors and activate endosomal TLR7/9 signaling. TLR expression is notably upregulated in peripheral blood mononuclear cells (PBMCs) from patients, highlighting engagement of this pathway (91). TLR signaling initiates the MyD88–IRF7 cascade, resulting in high levels of IFN-α, particularly in rapidly progressive ILD cases (92, 93). The initial IFN-α burst primes monocytes and macrophages by upregulating MDA5 and RIG-I, rendering them hypersensitive to extracellular RNA. These cells adopt a pathogenic phenotype and sustain inflammation by producing IFN-β through MAVS–IRF3/7 signaling (94, 95).

In MDA5+ DM-ILD patients, key chemokines like CCL2 and CXCL10 are upregulated, facilitating the recruitment of CD8+ T-cells, B-cells, and macrophages to pulmonary tissues (91, 96). Both T and B-cells in these patients express elevated levels of ISGs. For instance, CD8+ T-cells upregulate Granzyme K, and B-cells show enhanced survival and autoantibody production, likely via IFN-I induced BAFF signaling (97). Furthermore, lung biopsies stained for MxA, a highly IFN-I induced protein, indicate strong perivascular IFN-I activity. This perivascular IFN-I response is accompanied by increased adhesion molecule expression and markers of endothelial injury (91).

Importantly, high IFN-I signatures correlate with elevated ferritin, CRP, and myositis disease activity assessment Tool (MYOACT) scores, reflecting increased disease severity and worse prognosis (98).

Despite the aggressive nature of anti-MDA5+ ILD, patients show relatively mild fibrosis compared to other CTD-ILDs. This may be due to IFN-I driven upregulation of MDA5 in lung fibroblasts, which has been shown to suppress TGF-β signaling. In fibroblast cultures, IFN-I exposure induces MDA5 expression, which downregulates fibrotic markers including collagen I/III, α-SMA and connective tissue growth factor (CTGF) (99, 100). Clinically, this correlates with fewer fibrotic HRCT findings. Thus, MDA5 positivity, and the IFN-I environment that drives it, may protect against fibrosis, contributing to a distinct inflammatory, rather than fibrotic, ILD phenotype. Theoretically, this poses a possible risk of fibrotic exacerbation upon blockade of IFN-I signaling in patients. As IFN-I might be implicated in fibrosis in CTD-ILD, there might also be a role for IFN-I in PPF. Though different from PPF in a pathophysiological sense, looking at IFN-I in IPF sheds light at the role of IFN-I in PPF as reduced IFN-I signaling has been implicated in IPF. Common variants in IFN-I regulated genes that lead to decreased IFN-I signaling are associated with increased IPF risk and worse prognosis, implying that defects in antiviral signaling modulate how environmental insults like viral infections shape fibrotic progression (101). Further insight comes from *ELMOD2*, a candidate gene for familial IPF. In familial IPF, *ELMOD2* expression is reduced in pulmonary tissue. *ELMOD2* is expressed in alveolar epithelial cells and macrophages and plays a role in innate immune regulation. Overexpression of *ELMOD2* enhances IFN-I and IFN-III production in response to TLR3 activation whereas silencing impairs interferon responses. Interestingly, influenza A virus has been shown to downregulate *ELMOD2* expression, suggesting a viral evasion mechanism that may compromise epithelial integrity. These findings suggest that reduced *ELMOD2* expression in familial IPF may impair antiviral defense mechanisms, particularly interferon responses, contributing to increased susceptibility to viral injury and fibrotic disease (102).

However, IFN-I pathway involvement in IPF is likely heterogeneous. Single-cell RNA sequencing reveals muted ISG expression in most cell types, yet heightened IFN-I signatures in specific cell populations such as aberrant basaloid cells and distal airway epithelium, suggesting compartmentalized activation (103).

Taken together, these findings indicate a muted, dysfunctional, or spatially restricted IFN-I signaling in IPF, in contrast to heightened systemic IFN-I responses characteristics of CTD-ILDs. This may reflect a failure to mount effective antiviral responses, ultimately enabling chronic injury and fibrosis. Of note, IFN-II (IFN-γ) has already been tested as an antifibrotic therapy in IPF, but large randomized trials did not show clinical benefit, arguing against IFN-γ replacement in this setting (104). As IFN-I and IFN-II differ fundamentally in terms of cellular source (pDC/monocytes vs Th1/NK), receptor usage (IFNAR vs IFNGR) and downstream targets (STAT1/STAT2 heterodimer vs STAT1 homodimers) further investigation of IFN-I is warranted.

Of note, IFN-I does not only play a role in anti-MDA5+ ILD as is explored by a recent single cell characterization of anti-synthetase syndrome (ASS) ILD (105). Here, the authors report upregulated IFN-I responses in ASS-ILD although they do not correlate this to any radiological findings.

### IFN-I in Anti-Scl70 Positive SSc-ILD

SSc is characterized by widespread immune activation, vasculopathy, and fibrosis. Among its complications, ILD remains the leading cause of mortality. Elevated IFN-I activity, either measured as serum IFN-α protein levels or as whole blood qPCR ISG expression, is commonly observed in SSc and is implicated in ILD, especially in patients positive for anti-Scl70 antibodies (106, 107).

However, these findings remain the subject of debate. A recent commentary (108) questioned the robustness of these associations in one of the larger studies, pointing to methodological issues including cohort heterogeneity and limitations of the IFN-α2a assay (106). The commentary cautions against interpreting serum IFN-I levels as definitive ILD markers and advocates for further analyses. This underscores the complexity of IFN-I biology in SSc-ILD and the need for further investigation.

Despite this, transcriptomic and single-cell studies support a role for IFN-I in SSc-ILD. IFN-I signaling is elevated in myeloid and lymphoid cells in SSc-ILD. While absent in healthy lungs, pDCs appear in SSc-ILD pulmonary tissue and display activated IFN-I-related pathways (109).

Moreover, lung fibroblasts from SSc patients exhibit cGAS driven IFN-I secretion. Inhibition of cGAS in both *ex vivo* tissues and animal models reduces IFN-I production and fibrotic markers, suggesting therapeutic potential (86).

Clinically, high IFN-I induced chemokine scores are associated with ILD severity and progression, particularly in limited cutaneous SSc (110). This composite score is derived from serum levels of IFN-I-inducible chemokines including CCL2, CCL8, CCL19, CXCL9, CXCL10, and CXCL11 and serves as a surrogate marker for IFN-I activity. Elevated scores were associated with faster disease progression and performed well as a stratification tool.

## Clinical implications of IFN-I in ILD

Based on the available evidence, assessing IFN-I or its surrogate markers may provide additional clinical value for the diagnosis, stratification, and monitoring of patients with CTD-ILD. However, no standardized approach currently exists for measuring IFN-I pathway activation, and a large variety of assays are used (111). Harmonization and standardization of IFN-I pathway assessment are therefore needed.

Three complementary strategies are under evaluation to dampen IFN-I activity in CTD-ILD. At the receptor level, anifrolumab is being tested for SSc-ILD in the phase-3 DAISY trial. No efficacy or safety data from DAISY have been released as of yet, but current expectations rest on favorable safety profiles seen in SLE (35).

Further downstream, small uncontrolled case-series suggest that the JAKis tofacitinib and baricitinib can stabilize or modestly improve lung function in (refractory) anti-MDA5+ DM-ILD and SSc-ILD (112–114). In these studies, JAKis were well tolerated with reactivation of herpes zoster being the main concern.

Apart from patient studies, in vitro studies also underline a role for IFN-I modulating therapies. Inhibition of cGAS-STING reduces IFN-I signaling and expression of inflammatory and fibrotic mediators in various mouse models of pulmonary injury (radiation induced, autoimmune) (115, 116). Downstream of cGAS-STING, pre-clinical work shows that amlexanox, a blocker of TBK1, inhibits production of IFN-I and subsequent expression of ISGs in PBMCs from SjDs, SLE and SSc patients (117). Functionally, this translated into decreased B-cell differentiation, proliferation and IgG/IgM production (97).

Collectively, IFNAR blockade, JAK inhibition and cGAS–STING antagonists outline therapeutic strategies whose impact on ILD still awaits confirmation in further research. Future studies on IFN-I blocking/modulating therapies should also provide clarity on the possible antifibrotic effects of IFN-I and whether blocking IFN-I exacerbates fibrosis. The largest studies on IFN-I modulating therapies in humans are summarized in **Table 2**.

**Table 2:** Summary of patient studies concerning IFN-I modulating therapies in CTD-ILD.

Drug	CTD-ILD	Study design	Pulmonary endpoint(s)	Major findings	Reference
Tofacitinib	Anti-MDA5 DM–ILD	Cohort study of newly diagnosed anti-MDA5 DM-ILD receiving either calcinurininhibitor or tofacitinib	1-yr transplant-free survival	Better survival with tofacitinib (HR 0.72, 95% CI 0.56-0.94; p=0.013)	Wu et al. Eur Respir J 2025 PMID: 39884766 (12)
Tofacitinib	Anti-MDA5 DM–ILD	Meta-analysis of 4 cohorts of patients receiving tofacitinib (n=57) vs conventional therapy (glucocorticoids, mycophenolate mofetil, cyclosporine, n=90)	All-cause mortality	Reduced all-cause mortality (RR 0.61, 95% CI 0.41-0.91)	Wang et al. Ther Adv Respir Dis 2024 PMID: 39480695
Tofacitinib	SSc–ILD	Retrospective cohort study of conventional therapy + tofacitinib (n=9) vs conventional therapy alone (n=35)	DLCO, HRCT fibrosis score	Smaller decrease in DLCO in the tofacitinib treated group after 6 months (62.05 ± 9.47 vs. 66.61 ± 12.39, p=0.046), reduced HRCT scores of pulmonary fibrosis in tofacitinib treated patients (15.00 ± 3.87 vs. 12.66 ± 4.92, p=0.009)	Junfei et al. Clin Rheumatol 2023 PMID: 37335409 (114)
Tofacitinib ^II^	SjDs–ILD	Prospective RCT protocol, tofacitinib vs cyclofosfamide/azathioprine	FVC at 52 wks (primary)	Results pending	Gao et al. BMC Pulm Med 2023 PMID: 38007449
Anifrolumab	SSc-ILD	RCT in which patients receive either anifrolumab or a placebo alongside their conventional therapy	FVC (secondary)	Results pending	Khanna et al. Ann Rheum Dis 2022 PMID: 39152751

Khanna et al. – ^I^ - Study Details | NCT05925803 | Determine Effectiveness of Anifrolumab In SYstemic Sclerosis (DAISY) | ClinicalTrials.gov.

Gao et al. – ^II^ - ChiCTR2000031389, Version V1.1, version creation time 2020/03/29 23:14:12, Chinese Clinical Trial Registry.

## Discussion

IFN-I signaling is increasingly recognized as a key factor in the pathogenesis of several CTD-ILDs, acting both as a pathogenic mediator and a potential therapeutic target. Genetic, patient-based, and cellular studies consistently demonstrate its involvement with elevated IFN-I signatures correlating with disease severity, particularly in anti-MDA5+ DM-ILD and SSc-ILD. These signatures hold promise as prognostic biomarkers, though longitudinal studies are required to validate their predictive value. Despite these advances, important questions remain. CTD-ILD also occurs in patients without detectable IFN-I pathway activation, raising the possibility that IFN-I may exert a local effect in certain cases, or alternatively, plays no pathogenic role in some disease subsets. Furthermore, while the driving factor for increased IFN-I production is evident in patients with anti-Scl70 or anti-MDA5 autoantibodies, it remains unclear in those who are autoantibody negative yet display an elevated IFN-I signature. Before IFN-I based biomarkers or therapeutic strategies can be implemented clinically, larger trials and standardized measurements are needed to assess their diagnostic, therapeutic, and monitoring utility. Given that IFN-I comprises a family of 17 molecules, current assessments based on surrogate markers, such as ISG expression or IFN-I induced cytokines, should be rigorously compared across diseases and clinical contexts. Ultimately, ongoing trials targeting IFN-I pathways will be instrumental in determining the clinical benefits of pathway modulation, refining our ability to identify high-risk patients, and advancing personalized treatment strategies in CTD-ILD.
